# Downregulation of PHLPP induced by endoplasmic reticulum stress promotes eIF2α phosphorylation and chemoresistance in colon cancer

**DOI:** 10.1038/s41419-021-04251-0

**Published:** 2021-10-18

**Authors:** Bianqin Guo, Xiaopeng Xiong, Sumati Hasani, Yang-An Wen, Austin T. Li, Rebecca Martinez, Ashley T. Skaggs, Tianyan Gao

**Affiliations:** 1 Chongqing Key Laboratory of Translational Research for Cancer Metastasis and Individualized Treatment, Chongqing University Cancer Hospital, Chongqing, 400030 China; 2 Markey Cancer Center, University of Kentucky, Lexington, KY USA; 3 Department of Molecular and Cellular Biochemistry, University of Kentucky, Lexington, KY USA; 4 Paul Laurence Dunbar High School, Lexington, KY USA; 5 Agricultural and Medical Biotechnology Program, College of Agriculture, Food & Environment, University of Kentucky, Lexington, KY USA; 6 Present Address: Princeton University, Princeton, NJ USA

**Keywords:** Biochemistry, Cancer

## Abstract

Aberrant activation of endoplasmic reticulum (ER) stress by extrinsic and intrinsic factors contributes to tumorigenesis and resistance to chemotherapies in various cancer types. Our previous studies have shown that the downregulation of PHLPP, a novel family of Ser/Thr protein phosphatases, promotes tumor initiation, and progression. Here we investigated the functional interaction between the ER stress and PHLPP expression in colon cancer. We found that induction of ER stress significantly decreased the expression of PHLPP proteins through a proteasome-dependent mechanism. Knockdown of PHLPP increased the phosphorylation of eIF2α as well as the expression of autophagy-associated genes downstream of the eIF2α/ATF4 signaling pathway. In addition, results from immunoprecipitation experiments showed that PHLPP interacted with eIF2α and this interaction was enhanced by ER stress. Functionally, knockdown of PHLPP improved cell survival under ER stress conditions, whereas overexpression of a degradation-resistant mutant PHLPP1 had the opposite effect. Taken together, our studies identified ER stress as a novel mechanism that triggers PHLPP downregulation; and PHLPP-loss promotes chemoresistance by upregulating the eIF2α/ATF4 signaling axis in colon cancer cells.

## Introduction

The PHLPP family of phosphatases, consisting of PHLPP1 and PHLPP2 isoforms, belongs to the PPM superfamily of Ser/Thr protein phosphatases [1, 2]. Following the initial discovery of PHLPP as a protein phosphatase of AKT [3, 4], a number of studies have provided strong evidence supporting a pleiotropic role of PHLPP in inhibiting the initiation and progression of colon cancer by suppressing multiple oncogenic signaling pathways [5–9]. Consistent with a tumor suppressor role of PHLPP, loss of PHLPP expression was frequently identified in different cancer types, including leukemia, colorectal, pancreatic, and nonsmall cell lung cancer [7, 10–12], and lower PHLPP expression was found to be associated with increased malignancies and poor survival [10, 13, 14]. Moreover, studies on determining molecular mechanisms underlying PHLPP downregulation have identified the proteasome-dependent protein degradation as a major pathway leading to decreased PHLPP expression under cellular stress conditions, such as inflammation, hypoxia, and nutrient deprivation [15–17]. Although PHLPP-mediated regulation of AKT signaling has often been cited as a major underlying mechanism, how PHLPP-loss provides a survival advantage to cancer cells, especially under stress conditions, remains elusive.

Increased demand for ER-dependent protein synthesis, often driven by activation of oncogenic pathways, is needed to support cell growth and proliferation during tumorigenesis [18]. If the ER capacity to handle protein biogenesis is overwhelmed, improperly folded proteins accumulate in ER leading to a cellular state called ER stress [18, 19]. Reversible phosphorylation on Ser51 (S51) of eIF2α is a highly conserved regulatory event activated in response to ER stress. The phosphorylation of eIF2α reduces global translation but preferentially increases ATF4 translation, a master regulator controlling the transcription of key genes involved in the integrated stress response (ISR) [20]. Treatment with chemotherapy agents often induce ER stress. As a part of the ISR, the activation of eIF2α/ATF4 signaling axis is known to play a key role in the induction of autophagy, which contributes to the development of chemoresistance [18, 21]. Thus, a better understanding of molecular mechanisms that regulate ER stress and ISR is needed to improve the anticancer efficacy of standard chemotherapy drugs.

In this study, we determine the functional effect of PHLPP-loss on regulating chemosensitivity. We showed that chemotherapy-induced ER stress stimulated PHLPP downregulation by enhancing proteasome-mediated degradation. Knockdown of PHLPP increased the phosphorylation of eIF2α and promoted autophagy induction. Functionally, increased PHLPP expression was associated with improved responses to chemotherapy agents in colon cancer cells. Taken together, our findings demonstrate that PHLPP serves as a stress sensor to control chemosensitivity by regulating eIF2α-mediated ISR.

## Materials and methods

### Cells and reagents

Human colon cancer cell lines HCT116 and SW480 cells were cultured in McCoy’s 5A and DMEM supplemented with 10% fetal bovine serum (FBS, MilliporeSigma, MO, USA) and 1% penicillin-streptomycin, respectively. These cells were purchased from ATCC and authenticated using short tandem repeat (STR) DNA profiling (Genetica, OH, USA). Stable PHLPP1 and PHLPP2 knockdown cells were generated using lentivirus-based RNAi as described in previous studies [5, 7]. SW480 cells stably expressing vector, HA-PHLPP1 (a N-terminal truncated form of PHLPP1 originally termed PHLPP1α [1, 3]) or HA-PHLPP2 were described in previous studies [5]. To express WT PHLPP1 or a phosphorylation-deficient PHLPP1/4A (full-length PHLPP1 containing S1359A/T1363A/S1379A/S1381A mutations [22]) in HCT116 cells, the coding sequence of HA-PHLPP1 or HA-PHLPP1/4A was cloned into pBabe-puro retroviral vector. Tunicamycin, irinotecan, oxaliplatin, and MG-132 were purchased from MilliporeSigma.

### Immunoprecipitation and western blot analysis

Cells were harvested and detergent-solubilized cell lysates were obtained as described previously [5, 7, 8, 17]. Equal amounts of cell lysates were resolved by SDS-PAGE and subjected to western blot analysis. To examine the interaction between eIF2α and PHLPP, the solubilized cell lysates were incubated with either the anti-HA high-affinity antibody or the anti-PHLPP1 or PHLPP2 antibodies and protein A/G agarose beads (Thermo) at 4 °C for overnight. The beads were washed three times with lysis buffer and the immunoprecipitated proteins were analyzed by SDS-PAGE and Western blotting. The phospho-eIF2α, total eIF2α, ATF4, and LC3 antibodies were obtained from Cell Signaling. The PHLPP1 and PHLPP2 antibodies were from Bethyl laboratories. The β-actin and the anti-HA high-affinity antibodies were from MilliporeSigma.

### Real-time quantitative PCR (RT-qPCR)

Total RNA was isolated from human cancer cells using the PureLink RNA Mini Kit (Thermo, Fisher Scientific). Equal amounts of RNA (usually 100 ng of total RNA for each condition) were used as templates for the synthesis of cDNA using High Capacity cDNA Reverse Transcription kit (Thermo). The cDNAs obtained were combined with gene-specific primers listed in Supplementary Table S1 and SYBR Green Master Mix (Thermo). The final concentration of primers used was 500 nM. RT-qPCR reactions were carried out using QuantStudio 3 real-time PCR systems (Thermo). The relative gene expression was calculated based on the ΔΔCT method and all values were normalized to the level of β-actin.

### Drug treatment and cell viability assay

To determine cell viability in 2D culture, equal numbers of cells were seeded into 12-well plates. Subsequently, cells were treated with DMSO, tunicamycin (4 μg/ml), or irinotecan (10 μM) for 48 h in complete growth media. The cell viability was determined either by counting the number of live cells using an automated cell counter (Beckman-Coulter) or by staining with the alamarBlue Cell Viability Reagent (Thermo). The relative cell survival was calculated by normalizing numbers of surviving cells in the drug-treated group to that of the DMSO-treated group.

To determine cell viability in 3D culture, equal numbers of cells were mixed with 50% Matrigel in complete growth media and seeded into 24-well plates. Subsequently, cells were treated as described above for 48 h. The relative cell survival was determined using the CellTiter-Glo 3D viability assay (Promega) and normalized to the DMSO treatment group.

### Statistical analysis

Data from at least three independent experiments are expressed as means with SD as indicated in figure legends. Statistical analysis was performed using the Student *t* test for pairwise comparisons and one-way ANOVA for multiple comparisons. The relative mRNA expression results represent the average of three separate RT-qPCR experiments with four replicates for each gene in each experiment. For the Gene Set Enrichment Analysis (GSEA), RNA sequencing data were obtained from the TCGA COAD dataset. Correlations between the expressions of PHLPP and the other genes were quantified by Spearman’s correlation coefficient. The genes were then ordered from highest to lowest based on the correlation coefficient. This ranked list was inputted into the GSEA Desktop Application [23] to identify pathways that are associated with PHLPP expression.

## Results

### ER stress induces downregulation of PHLPP proteins via a proteasome-dependent mechanism in colon cancer cells

**Fig. 1:**
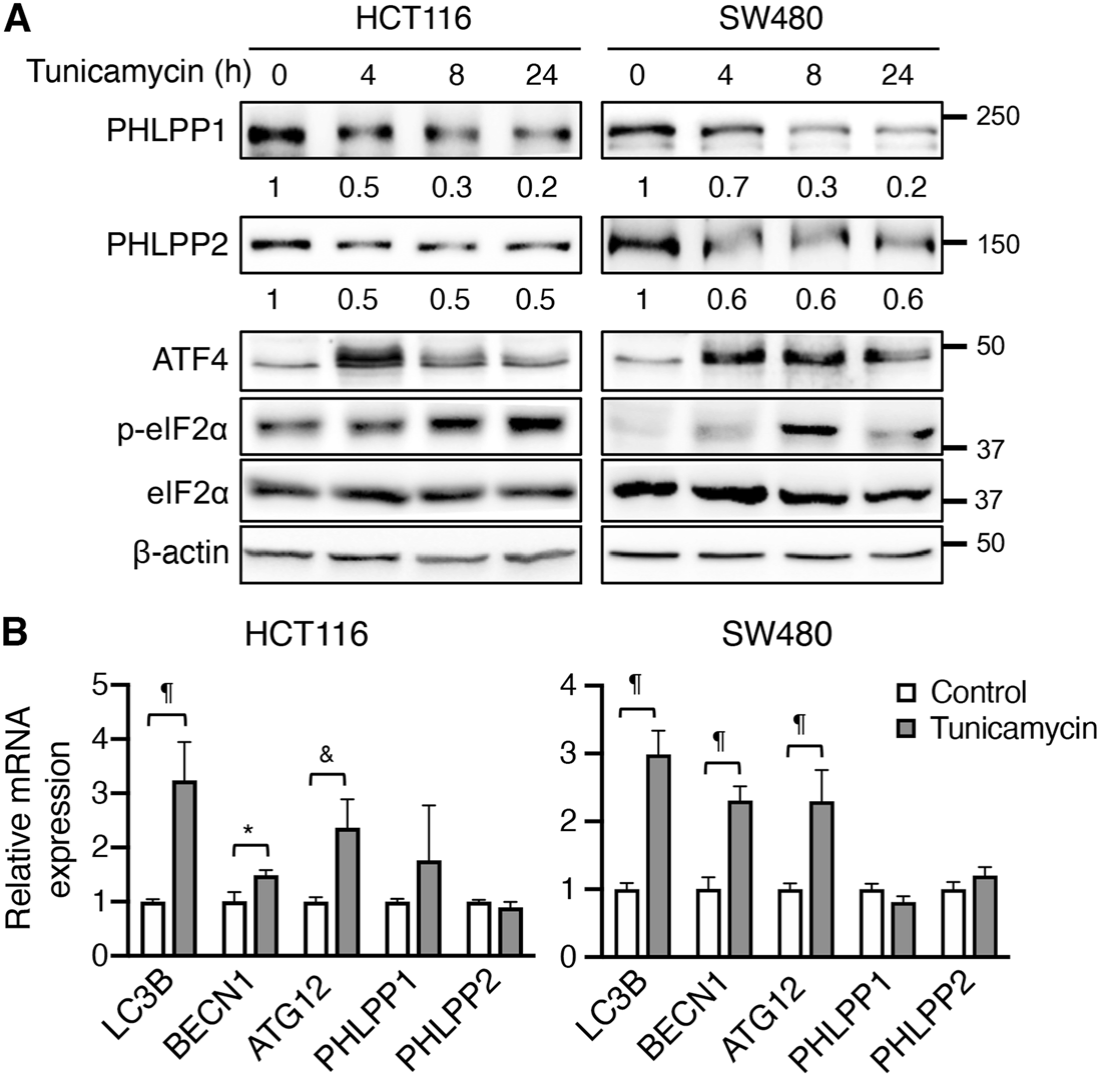
Tunicamycin-induced ER stress induces PHLPP downregulation. **a** HCT116 and SW480 cells were treated with tunicamycin for the indicated time. Cell lysates were analyzed for the expression of PHLPP1, PHLPP2, ATF4 and eIF2α and β-actin using Western blot. The phosphorylation status of eIF2α was detected using the phospho-eIF2α (p-eIF2α) antibody. The relative expression levels of PHLPP1 and PHLPP2 were obtained by normalizing to β-actin. **b** The relative expression of LC3B (gene name *MAP1LC3B*), *BECN1* (beclin1), *ATG12*, *PHLPP1*, and *PHLPP2* mRNA was determined using RT-qPCR following the treatment with tunicamycin for 24 h in HCT116 and SW480 cells. Data represent the mean ± SD (¶*p* < 0.0001, & *p* < 0.001 and *p < 0.05).

To determine the effect of ER stress on PHLPP expression, we treated colon cancer cells with tunicamycin, an ER stress inducer by inhibiting N-linked glycosylation. We found that tunicamycin treatment induced a time-dependent downregulation of both the PHLPP isoforms in HCT116 and SW480 cells. As expected, the expression of ATF4 and the phosphorylation of eIF2α were increased confirming the activation of ER stress (Fig. 1a). In addition, we analyzed the expression of autophagy-related genes downstream of ATF4 as well as PHLPP using RT-qPCR. Interestingly, the expression of PHLPP mRNAs was not significantly altered in cells treated with tunicamycin whereas ATF4 target genes, including LC3B (*MAP1LC3B*), beclin1 (*BECN1*), and *ATG12*, were induced by ER stress (Fig. 1b).

**Fig. 2:**
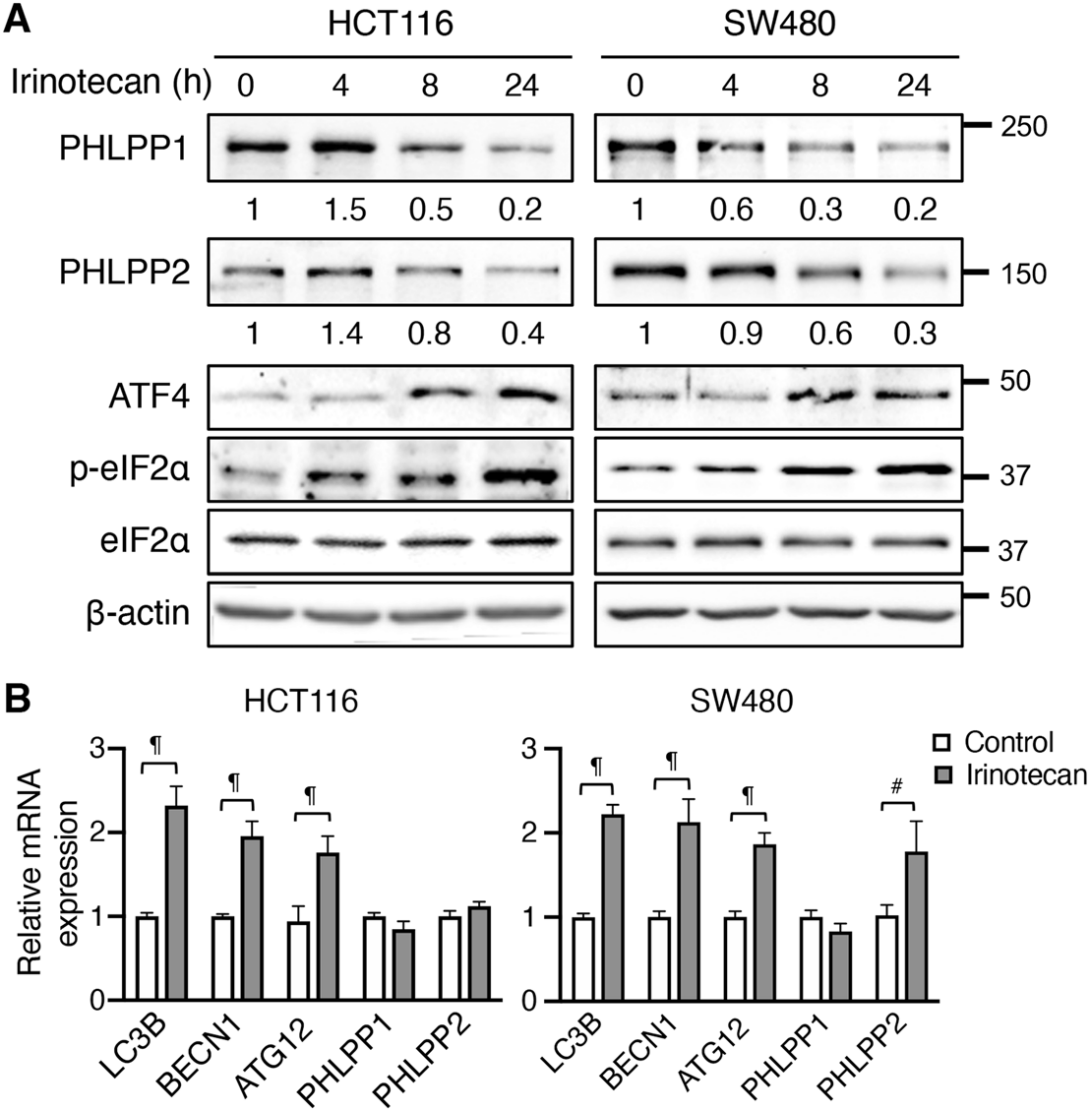
Chemotherapy drug treatment induces ER stress and PHLPP downregulation. **a** HCT116 and SW480 cells were treated with irinotecan for the indicated time. Cell lysates were analyzed for the expression of PHLPP1, PHLPP2, ATF4, p-eIF2α, eIF2α, and β-actin using Western blot. The relative expression levels of PHLPP1 and PHLPP2 were obtained by normalizing to β-actin. **b** The relative expression of *MAP1LC3B* (LC3B), *BECN1*, *ATG12*, *PHLPP1*, and *PHLPP2* mRNA was determined using RT-qPCR following the treatment with irinotecan for 24 h in HCT116 and SW480 cells. Data represent the mean ± SD (¶ *p* < 0.0001 and # *p* < 0.01).

We next treated HCT116 and SW480 cells with irinotecan, a clinically used chemotherapy drug in colon cancer. Consistently, the expression of both the PHLPP isoforms was decreased upon treatment, which coincided with increased phosphorylation of eIF2α and expression of ATF4 suggesting activation of ER stress (Fig. 2a). This decrease of PHLPP proteins was not due to the decrease of mRNA expression as results from RT-qPCR analysis revealed the expression of PHLPP mRNAs remained unchanged or increased (Fig. 2b). The expression of ATF4 target genes, including *MAP1LC3B*, *BECN1*, and *ATG12*, was increased confirming the induction of eIF2α/ATF4-dependent ISR (Fig. 2b). Similar downregulation of PHLPP proteins was observed in HCT116 and SW480 cells treated with oxaliplatin, another commonly used chemotherapy drug, as ER stress was induced in these cells (Supplemental Fig. S1).

**Fig. 3:**
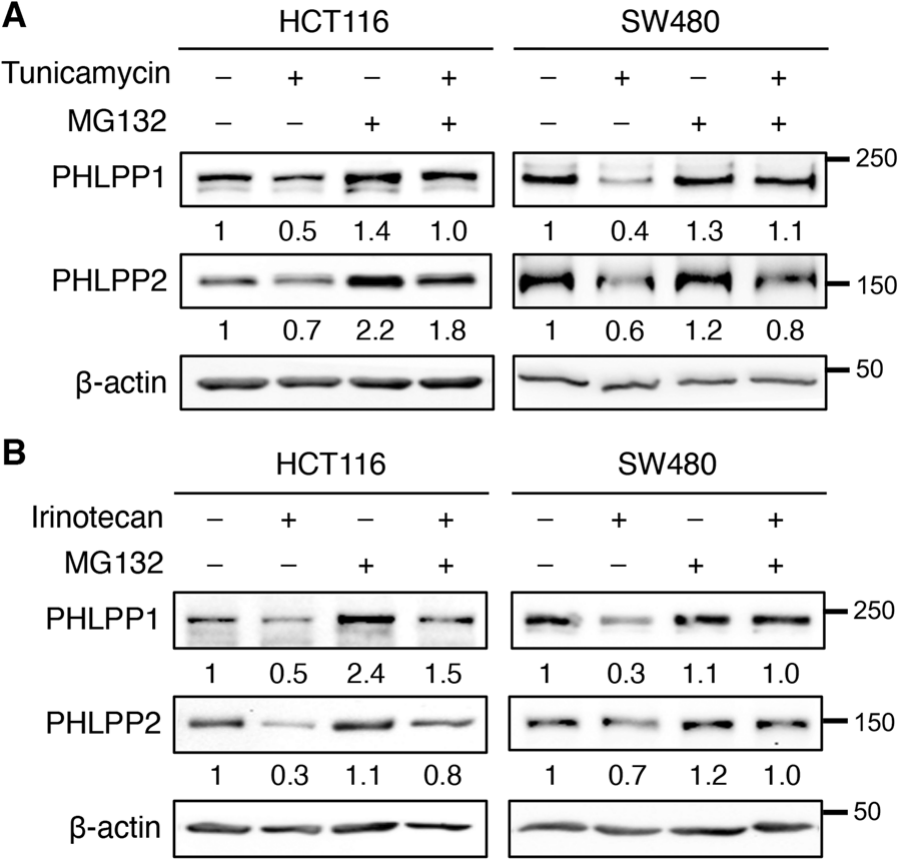
Inhibition of proteasome attenuates ER stress-induced PHLPP degradation in colon cancer cells. **a** HCT116 and SW480 cells were treated with tunicamycin for 24 h and MG-132 (10 μM) was added during the last 8 h of the treatment. The expression of PHLPP1, PHLPP2, and β-actin was analyzed using Western blot. The relative expression levels of PHLPP1 and PHLPP2 were obtained by normalizing to β-actin. **b** HCT116 and SW480 cells were treated with irinotecan for 24 h and MG-132 was added during the last 8 h of the treatment. The expression of PHLPP1, PHLPP2, and β-actin was analyzed using Western blot. The relative expression levels of PHLPP1 and PHLPP2 were obtained by normalizing to β-actin.

Since the expression of PHLPP mRNAs was not decreased upon the induction of ER stress, we treat cells with proteasome inhibitor MG-132 to determine if PHLPP downregulation is mediated via proteasome degradation. Indeed, both tunicamycin- and irinotecan-induced PHLPP downregulation was partially rescued by MG-132 treatment (Fig. 3), suggesting that ER stress promotes proteasome-dependent degradation of PHLPP. Additionally, we found that MG-132 treatment largely prevented the degradation of PHLPP proteins induced by oxaliplatin (Supplemental Fig. S2). Taken together, our results identified chemotherapy-induced-ER stress as a new mechanism that promotes PHLPP downregulation through the proteasome.

### Knockdown of PHLPP potentiates ER stress-induced activation of eIF2α/ATF4 signaling in colon cancer cells

**Fig. 4:**
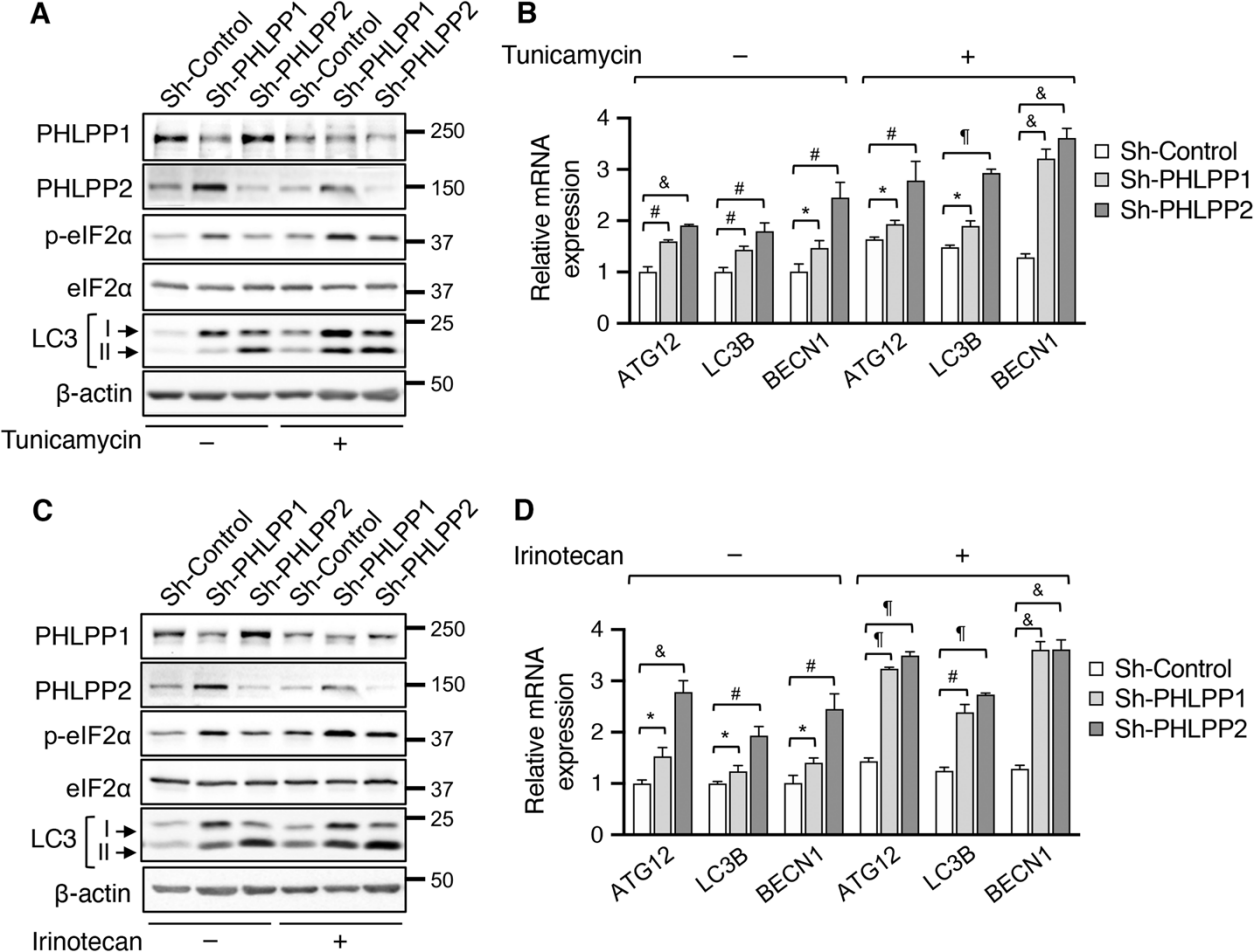
Knockdown of PHLPP1 or PHLPP2 enhances ER stress-induced autophagy response in colon cancer cells. **a** Control (sh-Control) and PHLPP knockdown (sh-PHLPP1 and sh-PHLPP2) SW480 cells were treated with DMSO or tunicamycin for 12 h. Cell lysates were analyzed for the expression of PHLPP1, PHLPP2, p-eIF2α, eIF2α, LC3, and β-actin using Western blot. The position of LC3-I and LC3-II was marked by arrows. **b** The relative expression of *ATG12*, *MAP1LC3B* (LC3B) and *BECN1* mRNA in DMSO or tunicamycin-treated cells was determined using RT-qPCR. Data represent the mean ± SD (¶ *p* < 0.0001, & *p* < 0.001, # *p* < 0.01, and * *p* < 0.05). **c** Control (sh-Control) and PHLPP knockdown (sh-PHLPP1 and sh-PHLPP2) SW480 cells were treated with DMSO or irinotecan overnight. Cell lysates were analyzed for the expression of PHLPP1, PHLPP2, p-eIF2α, eIF2α, LC3, and β-actin using Western blot. **d** The relative expression of *ATG12*, *MAP1LC3B* (LC3B) and *BECN1* mRNA in DMSO or irinotecan treated cells was determined using RT-qPCR. Data represent the mean ± SD (¶ *p* < 0.0001, & *p* < 0.001, # *p* < 0.01, and * *p* < 0.05).

Since ER stress is known to activate autophagy downstream of the eIF2α/ATF4 signaling pathway, we determined the effects of PHLPP-loss on modulating the autophagy phenotypes. Interestingly, knockdown of either PHLPP isoform increased the levels of eIF2α phosphorylation both basally and in tunicamycin-treated SW480 cells (Fig. 4a). In addition, the levels of LC3-II were elevated in PHLPP knockdown cells compared to that of control cells under ER stress (Fig. 4a). As a result, the expression of ATF4 target genes, including *ATG12*, *MAP1LC3B*, and *BECN1*, was increased in PHLPP knockdown cells both basally and in tunicamycin-treated cells (Fig. 4b). Furthermore, we found that silencing PHLPP isoforms resulted in similar increases in the levels of p-eIF2α and LC3-II proteins, as well as the expression of ATF4 target genes following irinotecan treatment (Fig. 4c–d), indicating elevated ER stress responses in PHLPP knockdown cells. Given that ER stress is also known to activate IRE1 via a phosphorylation-dependent mechanism [24], we determined if PHLPP downregulation alters IRE1-dependent unconventional splicing of *XBP1* mRNA. Interestingly, while the levels of spliced XBP1 mRNA (*XBP1s*) were largely upregulated in tunicamycin-treated cells, knockdown of PHLPP isoforms did not potentiate the effect of IRE1 on promoting XBP1 splicing (Supplemental Fig. S3). Thus, PHLPP-mediated regulation of ISR is likely due to its ability to control eIF2α phosphorylation upon activation of ER stress.

**Fig. 5:**
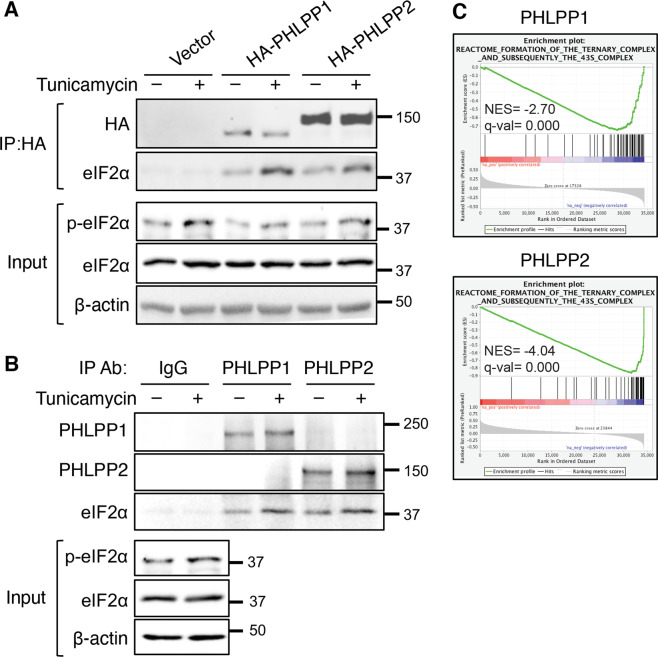
PHLPP interacts with eIF2α. **a** Stable SW480 cells expressing vector control, HA-PHLPP1 or HA-PHLPP2 were treated with DMSO or tunicamycin for 3 h. Cell lysates were immunoprecipitated using the anti-HA mAb. The presence of PHLPP and eIF2α in the immunoprecipitates was detected using the HA or eIF2α antibodies, respectively. The levels of p-eIF2α and total eIF2α in the input were analyzed using Western blot. **b** SW480 cells were treated with DMSO or tunicamycin for 3 h. Cell lysates were immunoprecipitated using rabbit IgG, anti-PHLPP1 or anti-PHLPP2 antibodies. The presence of PHLPP1, PHLPP2, and eIF2α in the immunoprecipitates was detected using the corresponding antibodies. **c** The Gene Set Enrichment Analysis (GSEA) was performed using the TCGA COAD RNA-seq dataset to identify genes that have positive or negative correlations with PHLPP1 and PHLPP expression. Enrichment plots showed significant correlation of the formation of the ternary complex and subsequently the 43S complex with the expression of PHLPP1 (NES = -2.70, FDR q-val = 0.0000) and PHLPP2 (NES = -4.04, FDR q-val = 0.0000) in colon cancer patients.

We next determine if PHLPP interacts with eIF2α to regulate its phosphorylation. To this end, the interaction between PHLPP isoforms and eIF2α was examined using co-immunoprecipitation experiments. Stable SW480 cells expressing vector, HA-PHLPP1 or HA-PHLPP2 were immunoprecipitated with the anti-HA antibody and endogenous eIF2α was found co-immunoprecipitated with both PHLPP isoforms (Fig. 5a). Interestingly, the amount of eIF2α interacting with PHLPP was increased upon tunicamycin treatment, suggesting that ER stress promotes the association between PHLPP and eIF2α (Fig. 5a). In addition, the levels of eIF2α phosphorylation were decreased in PHLPP overexpressing cells both basally and in tunicamycin-treated cells as shown in the input (Fig. 5a). Note that the HA-PHLPP1 protein expressed in SW480 cells was a N-terminal truncated form that originally named PHLPP1α in previous publications [25]. Since the N-terminal extension on PHLPP1 has recently been shown to regulate PHLPP-mediated cell cycle control [26], we analyzed the binding of the endogenous PHLPP1 (containing the N-terminal extension) and PHLPP2 with eIF2α. Consistently, the interaction between both the PHLPP isoforms and eIF2α was detected using co-immunoprecipitation experiments and the amount of eIF2α associated with PHLPP proteins was increased in cells treated with tunicamycin (Fig. 5b). To address if PHLPP regulates eIF2α phosphorylation at the level of PERK, co-immunoprecipitation experiments were conducted using antibodies against PHLPP. However, the interaction between the PHLPP and PERK was not detected (data not shown). In addition, we monitored PERK phosphorylation by detecting the appearance of higher molecular weight species upon the induction of ER stress. While knockdown of PHLPP increased eIF2α phosphorylation both the basically and upon tunicamycin treatment, decreased PHLPP expression had no effect on the pattern PERK phosphorylation (Supplemental Fig. S4). Together, these results suggest that PHLPP negatively regulates eIF2α phosphorylation through its interaction with eIF2α.

To support a functional connection between the PHLPP and eIF2α, the Gene Set Enrichment Analysis (GSEA) was performed using TCGA Colon Adenocarcinoma (COAD) dataset to identify gene sets that correlate with PHLPP1 and PHLPP2 expression [27–29]. Enrichment plots showed significant negative correlation of PHLPP1 (NES = –2.70, FDR q-val = 0.000) and PHLPP2 (NES = –4.04, FDR q-val = 0.000) with the “formation of the ternary complex and subsequently the 43s complex”, a process regulated by eIF2α phosphorylation (Fig. 5c). Collectively, our data suggested that PHLPP plays a role in negatively regulating the phosphorylation of eIF2α and ER stress-induced PHLPP degradation potentiates eIF2α/ATF4 signaling.

### Downregulation of PHLPP expression contributes to chemoresistance

**Fig. 6:**
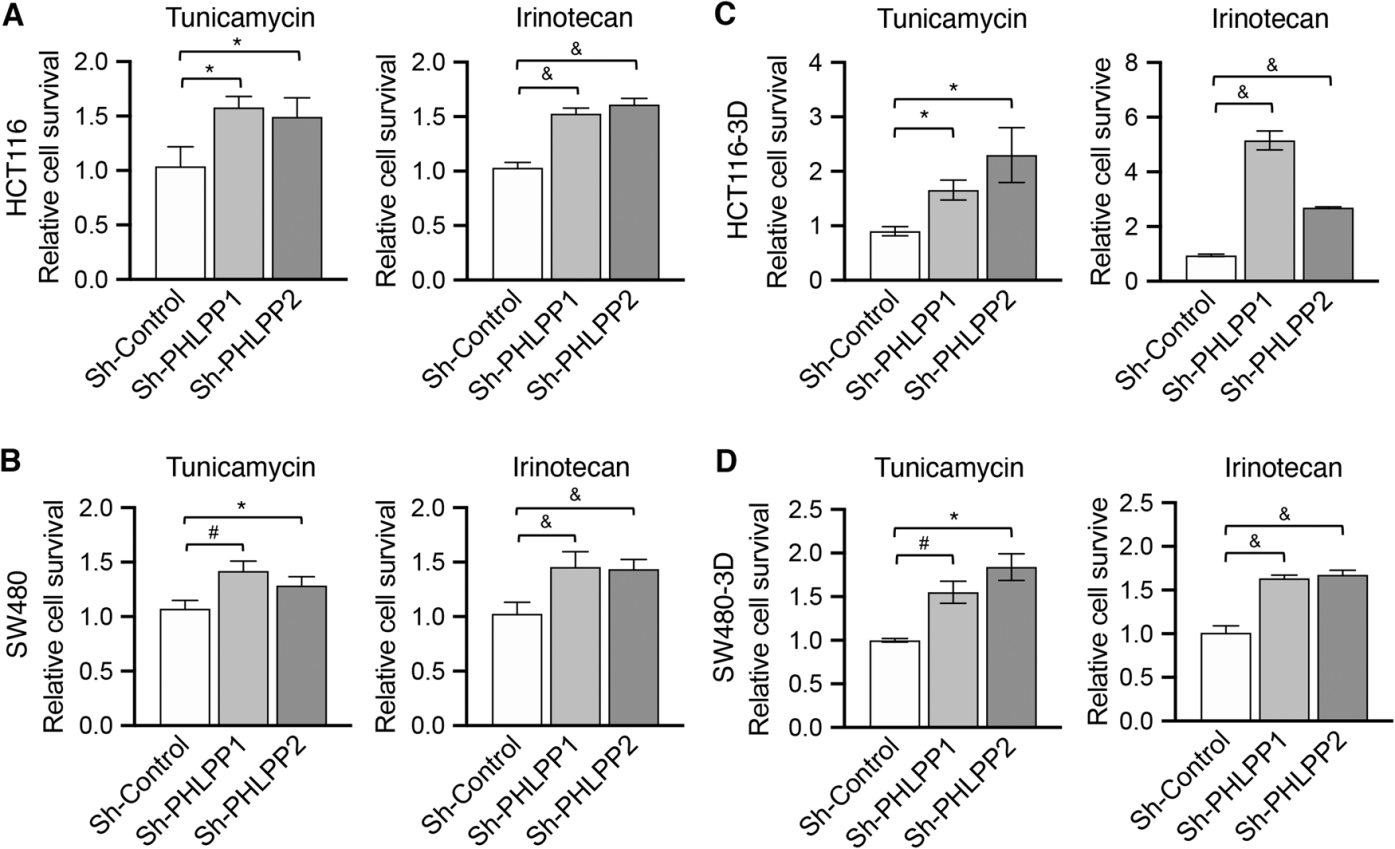
Downregulation of PHLPP promotes colon cancer cell survival. **a–b** Control (sh-Control) and PHLPP knockdown (sh-PHLPP1 and sh-PHLPP2) HCT116 (a) and SW480 (b) cells grown in 2D culture were treated with tunicamycin or irinotecan for 48 h. **c–d** Control (sh-Control) and PHLPP knockdown (sh-PHLPP1 and sh-PHLPP2) HCT116 (**c**) and SW480 cells (**d**) grown in 3D Matrigel were treated with tunicamycin or irinotecan for 48 h. The relative cell survival in PHLPP knockdown cells was normalized to sh-Control cells. Data shown in all bar graphs represent the mean ± SD (*n* = 3, & *p* < 0.001, # *p* < 0.01 and *p < 0.05).

As ER stress-induced upregulation of autophagy response is known to promote chemoresistance, we next determined if PHLPP expression regulates cell survival under ER stress conditions. Control and PHLPP knockdown HCT116 and SW480 cells were treated with tunicamycin or irinotecan and the numbers of cells survived were quantified. As shown in Fig. 6a, b, PHLPP knockdown cells were significantly more resistant to both tunicamycin- and irinotecan-induced cell death. Moreover, control and PHLPP knockdown colon cancer cells were cultured in 3D Matrigel and treated with tunicamycin or irinotecan. Similarly, silencing either PHLPP isoform rendered the cells more resistant to ER stress (Fig. 6c, d).

**Fig. 7:**
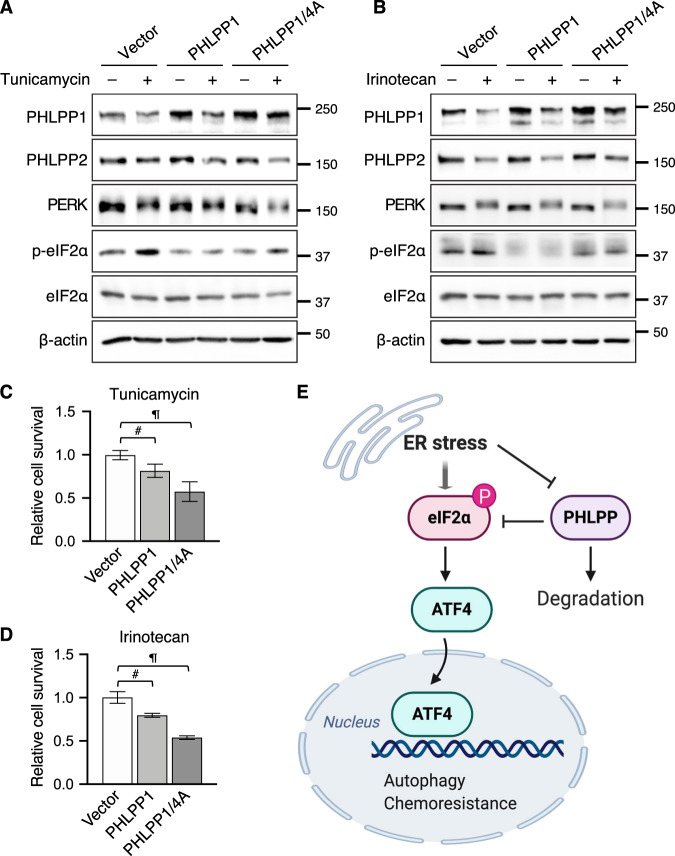
Overexpression of PHLPP decreases colon cancer cell survival. **a–b** HCT116 cells expressing either vector, PHLPP1 or PHLPP1/4A were treated with tunicamycin for 24 h (**a**) or irinotecan for 48 h (**b**). Cell lysates were analyzed for the expression of PHLPP1, PHLPP2, PERK, p-eIF2α, eIF2α, and β-actin using Western blot. **c–d** HCT116 cells expressing either vector, PHLPP1 or PHLPP1/4A were cultured 3D and treated with tunicamycin (**c**) or irinotecan (**d**) for 48 h. The relative cell survival was quantified and normalized to vector control cells. Data represent the mean ± SD (*n* = 3, # *p* < 0.01 and ¶ *p* < 0.0001). **e** A diagram shows that ER stress stimulates PHLPP degradation via a proteasome-dependent mechanism. This downregulation of PHLPP promotes the integrated stress response by enhancing eIF2α phosphorylation and ATF4-mediated activation of autophagy. Thus, PHLPP-loss represents a new mechanism underlying chemoresistance in colon cancer.

Furthermore, we determined if increased levels of PHLPP expression enhance chemosensitivity. To this end, HCT116 cells were transfected with a control vector, full-length PHLPP1 or PHLPP1/4A, a phosphorylation-deficient mutant PHLPP1. Our previous studies have identified four phosphorylation sites within the phosphatase domain of PHLPP1 that facilitate the proteasome-dependent degradation and mutating all four Ser and Thr residues to Ala (PHLPP1/4A) increases protein stability [22]. To determine relative cell survival, HCT116 cells expressing vector, PHLPP1, and PHLPP1/4A were cultured in 3D Matrigel and subjected to tunicamycin or irinotecan treatment. Interestingly, higher levels of both WT PHLPP1 and PHLPP1/4A mutant remained in cells treated with tunicamycin or irinotecan when compared to control cells; and PHLPP1/4A mutant proteins were relatively more resistant to ER stress-induced degradation (Fig. 7a, b). As controls, the degradation of endogenous PHLPP2 was not affected by the overexpression of PHLPP1 proteins. In addition, overexpression of PHLPP1 and PHLPP1/4A decreased eIF2α phosphorylation but had no effect on the appearance of PERK phosphorylated species (Fig. 7a, b). Functionally, increasing levels of PHLPP1 significantly decreased cell survival in both PHLPP1 and PHLPP1/4A expressing cells indicating enhanced sensitivity to both the tunicamycin and irinotecan. Consistent with increased protein stability, PHLPP1/4A mutant was more effective at decreasing cell survival (Fig. 7c, d). In addition, overexpression of PHLPP1 or PHLPP2 also rendered cells more sensitive to ER stress in SW480 cells (Supplemental Fig. S5). Taken together, results from our study showed that the expression of PHLPP is sensitive to ER stress-induced protein degradation; and loss of PHLPP promotes the activation of eIF2α/ATF4 signaling through increased phosphorylation of eIF2α. As a consequence, PHLPP downregulation provides a pro-survival advantage for cancer cells under cellular stress conditions (Fig. 7e).

## Discussion

It has been widely recognized that ER stress-induced unfolded protein responses are commonly associated with solid tumors [18, 30]. Both tumor intrinsic factors and conditions presented by the tumor microenvironment can lead to the activation of ER stress that controls the balance of apoptosis and cell survival. In particular, the induction of ER stress-mediated ISR downstream of eIF2α/ATF4 signaling has been identified as one of the major mechanisms contributing to chemotherapy resistance [18]. In our efforts to determine the mechanisms by which the expression of PHLPP proteins is downregulated in colon cancer, we found that ER stress stimulates proteasome-mediated PHLPP degradation. However, since MG-132 treatment only partially rescues PHLPP expression and a previously identified ubiquitination-resistant PHLPP1/4A mutant still undergoes delayed degradation under ER stress conditions, future studies are needed to determine if ER stress also inhibits the translation of PHLPP proteins. Mechanistically, downregulation of PHLPP potentiates eIF2α/ATF4-mediated autophagy response as a result of increased S51 phosphorylation of eIF2α. Consistently, our informatic analysis of TCGA COAD dataset revealed that PHLPP expression is negatively associated with the formation of the preinitiation complex, an eIF2α phosphorylation-dependent process required for protein translation initiation. To define the functional significance of PHLPP-loss, we demonstrated that silencing PHLPP expression renders cancer cells resistant to chemotherapy drugs whereas overexpression of PHLPP increases chemosensitivity. Together, our study identifies a novel role of PHLPP in regulating cell survival under ER stress conditions.

Our previous studies have shown that cellular stress conditions, such as hypoxia and nutrient deprivation [16, 17], can lead to PHLPP downregulation by promoting proteasome-dependent degradation. This stress-induced PHLPP-loss activates cell survival pathways downstream of AKT/mTOR signaling [7, 16, 17]. Here we identified ER stress as another tumor-associated stress signal that stimulates PHLPP degradation to potentiate eIF2α/ATF4 signaling. Since solid tumors are known to rely on ER stress-mediated IRS for tumor initiation and progression [18, 30], our findings suggested that this persistent ER stress may lead to the loss of PHLPP phenotype frequently observed in colon and other cancer types. Given that the expression of PHLPP proteins can be downregulated by various stress conditions, we reasoned that this post-translationally controlled mechanism triggers a rapid self-protective response to activate multiple pro-survival pathways. However, by degrading a pleiotropic tumor suppressor, cancer cells hijack this “first responder” reaction to drive tumorigenesis and chemoresistance. Consistently, reduced PHLPP expression has been associated with resistance to both cytotoxic- and targeted-therapies in other types of cancer [13, 16, 31, 32].

Furthermore, we showed that the expression of PHLPP controls the phosphorylation status of eIF2α at S51 site. Previous studies have identified GADD34- and CReP-containing PP1c complex as phosphatases of eIF2α; [20, 33, 34]; however, the effects of these PP1 complexes on eIF2α phosphorylation have only been examined in non-cancer cells and neither of the complexes has been implicated in regulating chemosensitivity. Since the expression of GADD34 increases whereas CReP remains unchanged during ER stress [33, 34], we reasoned that chemotherapy-induced PHLPP degradation represents a distinct mechanism to promote cell survival under ER stress by upregulating eIF2α phosphorylation. To support the notion that PHLPP functions as a new phosphatase of eIF2α, we showed in this study that the expression levels of PHLPP negatively correlate with eIF2α phosphorylation at S51 and the interaction between the endogenous PHLPP and eIF2α is readily detected by co-immunoprecipitation experiments. Interestingly, the induction of ER stress strengthens the interaction between PHLPP and eIF2α suggesting that PHLPP may be recruited to eIF2α as a negatively feedback mechanism to suppress prolonged inhibition of eIF2α. However, our results do not rule out the possibility that PHLPP-mediated dephosphorylation is indirect. Further characterization how ER stress promotes the direct interaction between PHLPP and eIF2α will help better understand the role of PHLPP as a phosphatase toward eIF2α. Functionally, silencing PHLPP potentiates ER stress-induced autophagy as indicated by increased levels of autophagy-related genes downstream of ATF4 and higher levels of LC3-II expression. In contrast to our finding that decreased PHLPP expression potentiates ER stress-induced autophagy, it has been shown previously that knockdown of PHLPP1 attenuates the chaperone-mediated autophagy by enhancing lysosomal Akt/mTOR activation [35]. It is likely that the functional interplay between PHLPP and autophagy can be differentially controlled by upstream signaling. Moreover, a recent study demonstrated that the phosphorylation of eIF2α at S51 is required for a large numbers of structurally distinct pharmacological agents to induce autophagy regardless of upstream kinases involved [36]. Future studies are needed to determine if PHLPP-mediated regulation autophagy represents a common mechanism to control cell survival when eIF2α phosphorylation is induced.

In summary, results from our study identified ER stress as a new mechanism contributing to PHLPP downregulation in colon cancer. Given our findings that PHLPP-loss plays a pivotal role in orchestrating a multitude of pro-survival responses downstream of cellular stress signals, the levels of PHLPP expression may be used to predict the effectiveness of anticancer agents.

## Supplementary information

Supplemental figures

## Data Availability

The datasets generated and/or analyzed during the current study are available from the corresponding author on reasonable request.

## References

[1] Brognard J, Newton AC (2008). PHLiPPing the switch on Akt and protein kinase C signaling. Trends Endocrinol Metab.

[2] Grzechnik AT, Newton AC (2016). PHLPPing through history: a decade in the life of PHLPP phosphatases. Biochem Soc Trans.

[3] Gao T, Furnari F, Newton AC (2005). PHLPP: a phosphatase that directly dephosphorylates Akt, promotes apoptosis, and suppresses tumor growth. Mol Cell.

[4] Brognard J, Sierecki E, Gao T, Newton AC (2007). PHLPP and a second isoform, PHLPP2, differentially attenuate the amplitude of Akt signaling by regulating distinct Akt isoforms. Mol Cell.

[5] Li X, Stevens PD, Liu J, Yang H, Wang W, Wang C (2014). PHLPP is a negative regulator of RAF1, which reduces colorectal cancer cell motility and prevents tumor progression in mice. Gastroenterology.

[6] Liu J, Stevens PD, Li X, Schmidt MD, Gao T (2011). PHLPP-mediated dephosphorylation of S6K1 inhibits protein translation and cell growth. Mol Cell Biol.

[7] Liu J, Weiss HL, Rychahou P, Jackson LN, Evers BM, Gao T (2009). Loss of PHLPP expression in colon cancer: role in proliferation and tumorigenesis. Oncogene.

[8] Xiong X, Wen YA, Mitov MI, Oaks CM, Miyamoto S, Gao T (2017). PHLPP regulates hexokinase 2-dependent glucose metabolism in colon cancer cells. Cell Death Disco.

[9] Xiong X, Li X, Wen YA, Gao T (2016). Pleckstrin homology (PH) domain leucine-rich repeat protein phosphatase controls cell polarity by negatively regulating the activity of atypical protein kinase C. J Biol Chem.

[10] Wang H, Gu R, Tian F, Liu Y, Fan W, Xue G (2019). PHLPP2 as a novel metastatic and prognostic biomarker in non-small cell lung cancer patients. Thorac Cancer.

[11] Smith AJ, Wen YA, Stevens PD, Liu J, Wang C, Gao T (2016). PHLPP negatively regulates cell motility through inhibition of Akt activity and integrin expression in pancreatic cancer cells. Oncotarget.

[12] Suljagic M, Laurenti L, Tarnani M, Alam M, Malek SN, Efremov DG (2010). Reduced expression of the tumor suppressor PHLPP1 enhances the antiapoptotic B-cell receptor signal in chronic lymphocytic leukemia B-cells. Leukemia.

[13] Lv D, Yang H, Wang W, Xie Y, Hu W, Ye M (2015). High PHLPP expression is associated with better prognosis in patients with resected lung adenocarcinoma. BMC Cancer.

[14] Zhou J, Yu X, Wang J, Li T, Jin T, Lei D (2015). Aberrant expression of PHLPP1 and PHLPP2 correlates with poor prognosis in patients with hypopharyngeal squamous cell carcinoma. PLoS One.

[15] Wen YA, Li X, Goretsky T, Weiss HL, Barrett TA, Gao T (2015). Loss of PHLPP protects against colitis by inhibiting intestinal epithelial cell apoptosis. Biochim Biophys Acta.

[16] Liu J, Stevens PD, Gao T (2011). mTOR-dependent regulation of PHLPP expression controls the rapamycin sensitivity in cancer cells. J Biol Chem.

[17] Wen YA, Stevens PD, Gasser ML, Andrei R, Gao T (2013). Downregulation of PHLPP expression contributes to hypoxia-induced resistance to chemotherapy in colon cancer cells. Mol Cell Biol.

[18] Avril T, Vauleon E, Chevet E (2017). Endoplasmic reticulum stress signaling and chemotherapy resistance in solid cancers. Oncogenesis.

[19] Cubillos-Ruiz JR, Bettigole SE, Glimcher LH (2017). Tumorigenic and Immunosuppressive Effects of Endoplasmic Reticulum Stress in Cancer. Cell.

[20] Pakos-Zebrucka K, Koryga I, Mnich K, Ljujic M, Samali A, Gorman AM (2016). The integrated stress response. EMBO Rep..

[21] B’Chir W, Maurin AC, Carraro V, Averous J, Jousse C, Muranishi Y (2013). The eIF2alpha/ATF4 pathway is essential for stress-induced autophagy gene expression. Nucleic Acids Res.

[22] Li X, Liu J, Gao T (2009). beta-TrCP-mediated ubiquitination and degradation of PHLPP1 are negatively regulated by Akt. Mol Cell Biol.

[23] Subramanian A, Tamayo P, Mootha VK, Mukherjee S, Ebert BL, Gillette MA (2005). Gene set enrichment analysis: a knowledge-based approach for interpreting genome-wide expression profiles. Proc Natl Acad Sci USA.

[24] Gardner BM, Pincus D, Gotthardt K, Gallagher CM, Walter P (2013). Endoplasmic reticulum stress sensing in the unfolded protein response. Cold Spring Harb Perspect Biol.

[25] O’Neill AK, Niederst MJ, Newton AC. Suppression of survival signalling pathways by the phosphatase PHLPP. FEBS J. 2012;280:572–83. 10.1111/j.1742-4658.2012.08537.x PMC3770140 22340730

[26] Kawashima AT, Wong C, Lorden G, King CC, Lara-Gonzalez P, Desai A, et al. The PHLPP1 N-Terminal Extension is a Mitotic Cdk1 Substrate and Controls an Interactome Switch. Mol Cell Biol. 2021;41:e00333–20. 10.1128/MCB.00333-20 PMC8088274 33397691

[27] Stevens PD, Wen YA, Xiong X, Zaytseva YY, Li AT, Wang C (2018). Erbin Suppresses KSR1-Mediated RAS/RAF Signaling and Tumorigenesis in Colorectal Cancer. Cancer Res.

[28] Wen YA, Xiong X, Scott T, Li AT, Wang C, Weiss HL (2019). The mitochondrial retrograde signaling regulates Wnt signaling to promote tumorigenesis in colon cancer. Cell Death Differ.

[29] Wen YA, Xiong X, Zaytseva YY, Napier DL, Vallee E, Li AT (2018). Downregulation of SREBP inhibits tumor growth and initiation by altering cellular metabolism in colon cancer. Cell death Dis.

[30] Ma Y, Hendershot LM (2004). The role of the unfolded protein response in tumour development: friend or foe?. Nat Rev Cancer.

[31] Hou J, Wang L (2012). FKBP5 as a selection biomarker for gemcitabine and Akt inhibitors in treatment of pancreatic cancer. PLoS One.

[32] Shi H, Hugo W, Kong X, Hong A, Koya RC, Moriceau G (2014). Acquired resistance and clonal evolution in melanoma during BRAF inhibitor therapy. Cancer Disco.

[33] Reid DW, Tay AS, Sundaram JR, Lee IC, Chen Q, George SE (2016). Complementary roles of GADD34- and CReP-containing eukaryotic initiation factor 2alpha phosphatases during the unfolded protein response. Mol Cell Biol.

[34] Jousse C, Oyadomari S, Novoa I, Lu P, Zhang Y, Harding HP (2003). Inhibition of a constitutive translation initiation factor 2alpha phosphatase, CReP, promotes survival of stressed cells. J Cell Biol.

[35] Arias E, Koga H, Diaz A, Mocholi E, Patel B, Cuervo AM (2015). Lysosomal mTORC2/PHLPP1/Akt Regulate Chaperone-Mediated Autophagy. Mol Cell.

[36] Humeau J, Leduc M, Cerrato G, Loos F, Kepp O, Kroemer G (2020). Phosphorylation of eukaryotic initiation factor-2alpha (eIF2alpha) in autophagy. Cell death Dis.

