# Supplementary material for: Downregulation of PHLPP induced by endoplasmic reticulum stress promotes eIF2α phosphorylation and chemoresistance in colon cancer
Source: Cell Death Dis. 2021 Oct 18;12(11):960. doi: 10.1038/s41419-021-04251-0 (PMC8523518; doi:10.1038/s41419-021-04251-0)
Supplement: Supplementary file 1 — Supplemental figures [file 41419_2021_4251_MOESM1_ESM.docx]

**Downregulation of PHLPP induced by ER stress promotes eIF2α phosphorylation and autophagy in colon cancer**

**Supplemental Figures**

**Figure S1. Oxaliplatin treatment induces ER stress and PHLPP downregulation in colon cancer cells.** (**a**) HCT116 and SW480 cells were treated with oxaliplatin (10 μM) for the indicated time. Cell lysates were analyzed for the expression of PHLPP1, PHLPP2, ATF4, p-eIF2α, eIF2α and β-actin using Western blot. The relative expression levels of PHLPP1 and PHLPP2 were obtained by normalizing to β-actin. (**b**) The relative expression of LC3, BECN1, ATG12, PHLPP1 and PHLPP2 mRNA was determined using RT-PCR following the treatment with oxaliplatin for 24 h in HCT116 and SW480 cells. Data represent the mean ± SD (& p < 0.001, # p < 0.01 and * p < 0.05).


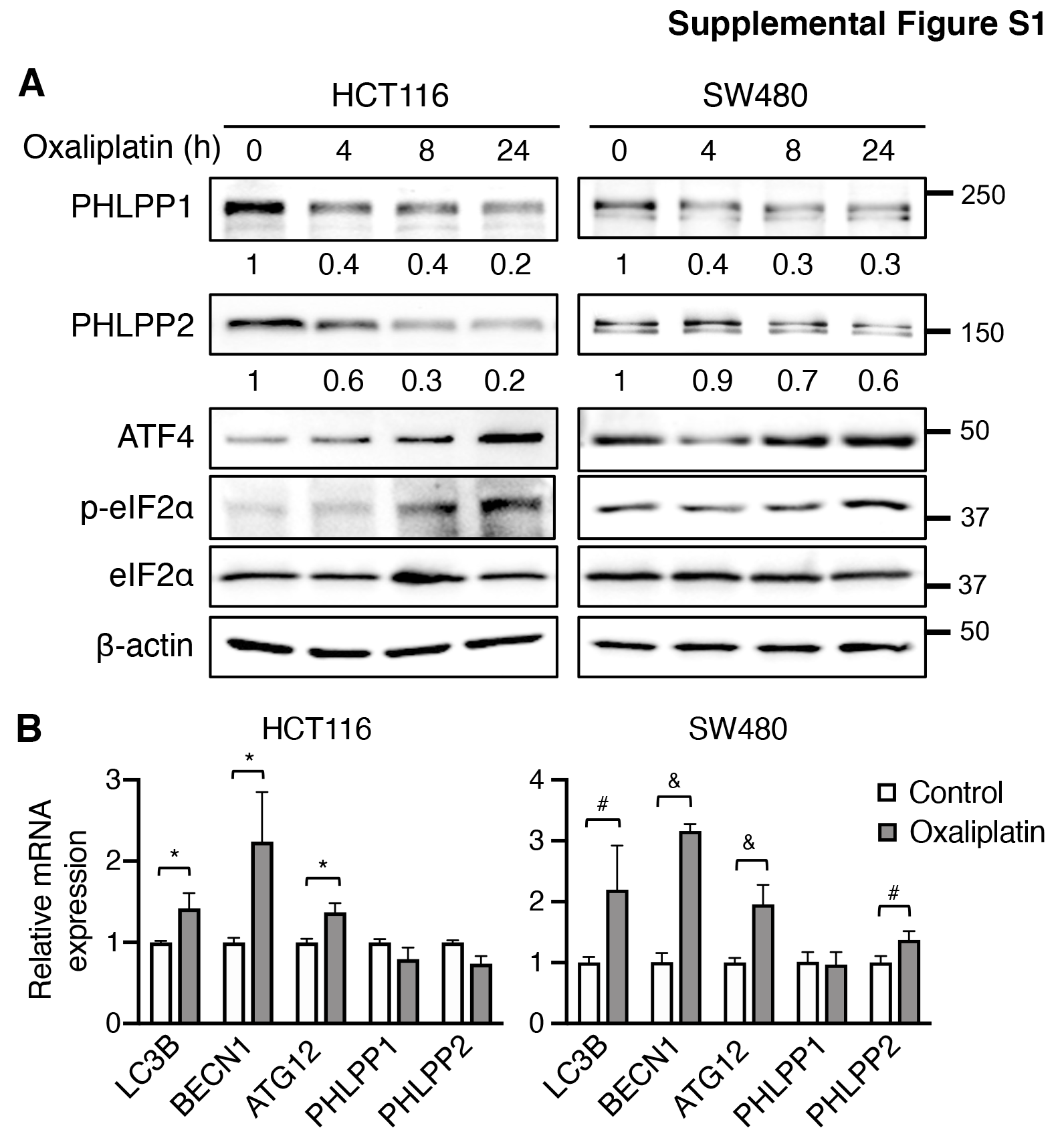


**Figure S2. Inhibition of proteasome-mediated protein degradation attenuates ER stress-induced PHLPP downregulation in colon cancer cells.** HCT116 and SW480 cells were treated with oxaliplatin for 24 h and MG-132 (10 μM) was added during the last 8 h of the treatment. The expression of PHLPP1, PHLPP2 and β-actin was analyzed using Western blot. The relative expression levels of PHLPP1 and PHLPP2 were obtained by normalizing to β-actin.


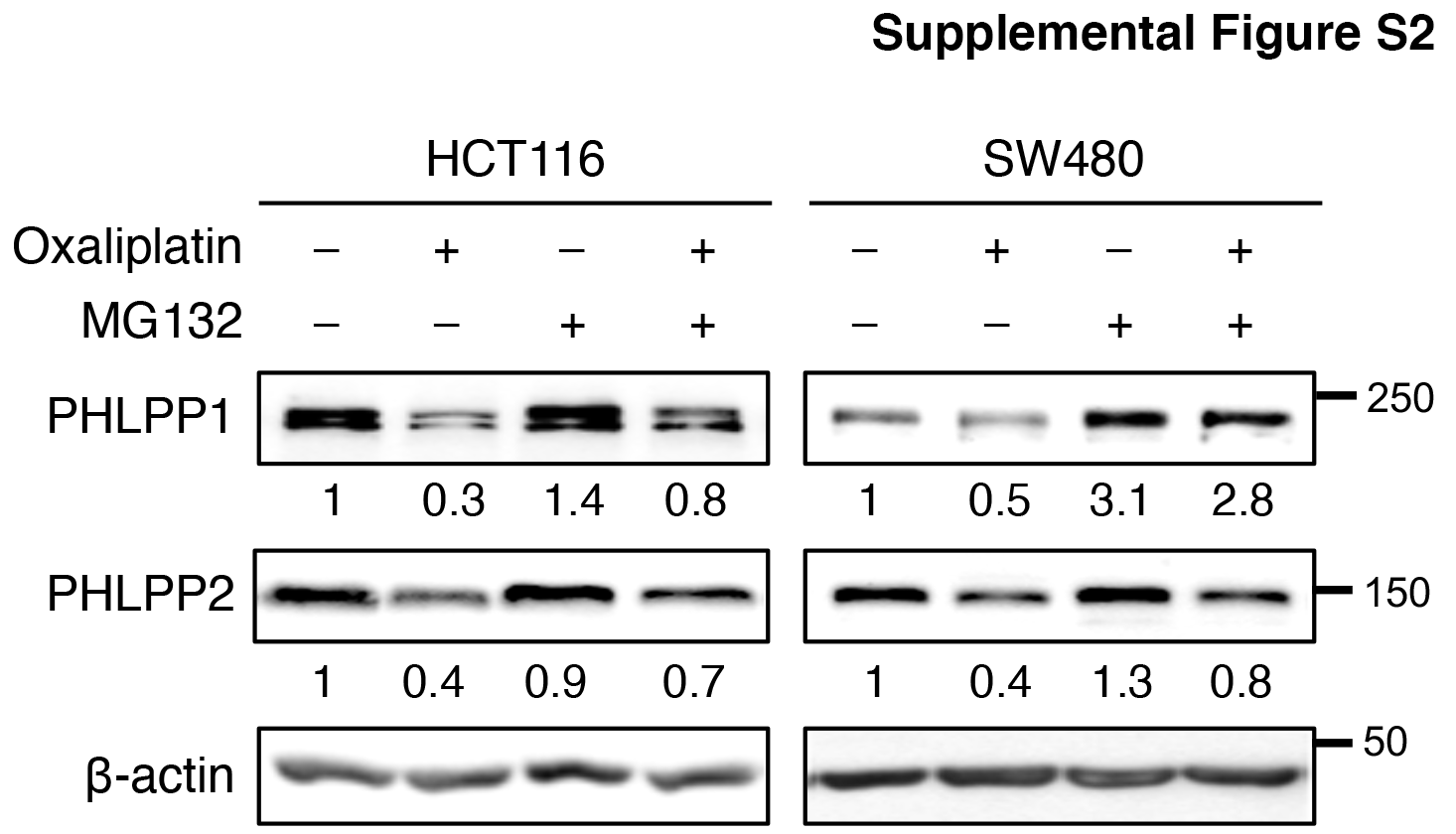


**Figure S3. The effect of PHLPP downregulation on ER stress-induced activation of IRE1-mediated XBP1 mRNA splicing.** Stable sh-control, sh-PHLPP1 and sh-PHLPP2 SW480 cells were treated with DMSO or tunicamycin for 24 h. The relative expression of XBP1s was analyzed using RT-qPCR. Data represent the mean ± SD (¶ p < 0.0001).

**Figure S4. The effect of silencing PHLPP on PERK phosphorylation.** Stable sh-control, sh-PHLPP1 and sh-PHLPP2 SW480 cells were treated with DMSO or tunicamycin for 16 h. Cell lysates were analyzed for the expression of PHLPP1, PHLPP2, PERK, p-eIF2α, eIF2α and β-actin using Western blot.

**Figure S5. Overexpression of WT PHLPP1 or PHLPP2 decrease colon cancer cell survival**. SW480 cells stably expressing vector, HA-PHLPP1 or HA-PHLPP2 were cultured in regular growth media and treated with tunicamycin or irinotecan for 24 h. The relative cell survival was quantified by counting number of live cells and normalized to vector control cells. Data represent the mean ± SD (n=3, # p < 0.01 and * p < 0.05).

**
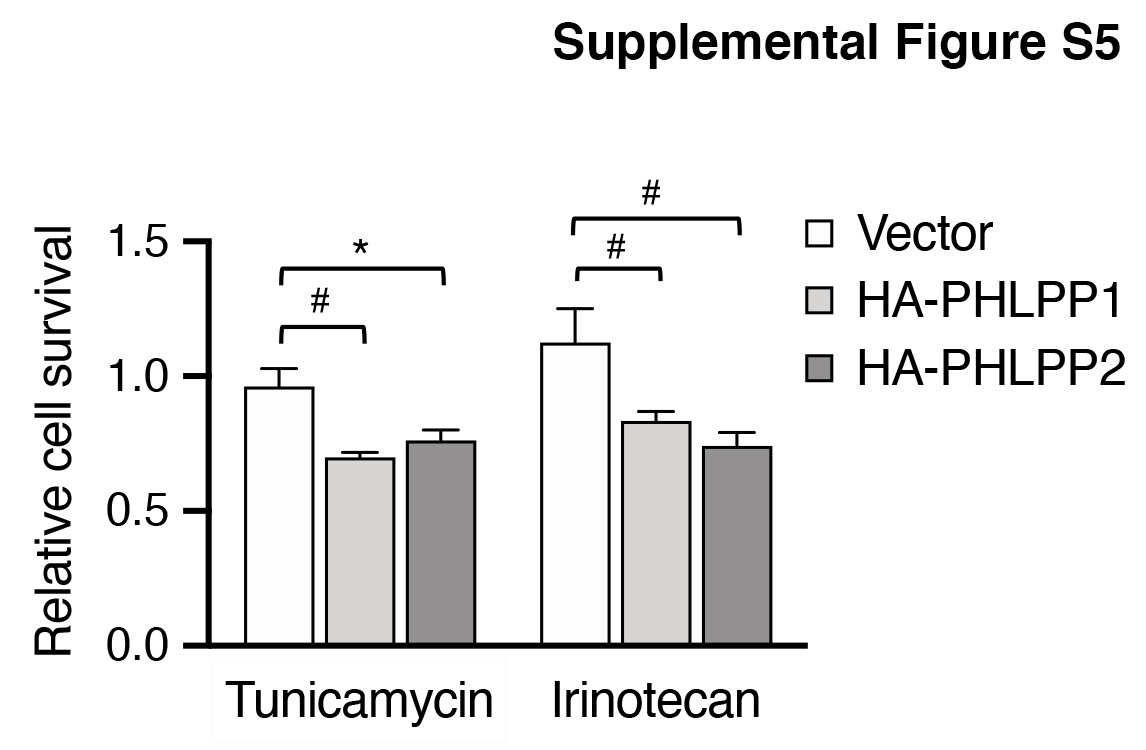
**

**Supplemental Table S1. Oligonucleotides used for RT-qPCR analysis**

**The gene specific PCR primers listed below were designed by PrimerBank if no specific reference was cited.**

| **Name** | **Forward (5’-3’)** | **Reverse (5’-3’)** |
| --- | --- | --- |
| *ACTB* | CATGTACGTTGCTATCCAGGC | CTCCTTAATGTCACGCACGAT |
| *ATG12* | CTGCTGGCGACACCAAGAAA | CGTGTTCGCTCTACTGCCC |
| *BECN1* | GGTGTCTCTCGCAGATTCATC | TCAGTCTTCGGCTGAGGTTCT |
| *MAP1LC3B (LC3B)* | AAGGCGCTTACAGCTCAATG | CTGGGAGGCATAGACCATGT |
| *PHLPP1* | GTTCTGCCACTAATTGGTGGA | GCTGGGATGCAACCTTGGA |
| *PHLPP2* | TGGAACCTACTGAACGACCTC | ATCCAAACGATCCATGTGGCA |
| *XBP1s ^1^* | TGCTGAGTCCGCAGCAGGTG | GCTGGCAGGCTCTGGGAAAG |

References:

1. Lhomond S*, et al.* Dual IRE1 RNase functions dictate glioblastoma development. *EMBO Mol Med* **10**, e7929 (2018)
